# c.1810C>T Polymorphism of *NTRK1 *Gene is associated with reduced Survival in Neuroblastoma Patients

**DOI:** 10.1186/1471-2407-9-436

**Published:** 2009-12-13

**Authors:** Beata S Lipska, Elżbieta Drożynska, Paola Scaruffi, Gian Paolo Tonini, Ewa Iżycka-Świeszewska, Szymon Ziętkiewicz, Anna Balcerska, Danuta Perek, Alicja Chybicka, Wojciech Biernat, Janusz Limon

**Affiliations:** 1 Department of Biology and Genetics, Medical University of Gdańsk, Dębinki 1, 80-211 Gdańsk, Poland; 2 Institute of Pediatrics, Medical University of Gdańsk, Dębinki 7, 80-211 Gdańsk, Poland; 3 Translational Pediatric Oncology, National Cancer Research Institute, L.go R. Benzi, 10 16132 Genoa, Italy; 4 Department of Pathology, Medical University of Gdańsk, Dębinki 7, 80-211 Gdańsk, Poland; 5 Department of Molecular and Cellular Biology, Intercollegiate Faculty of Biotechnology, University of Gdańsk, Kładki 24, 80-822 Gdańsk, Poland; 6 Department of Oncology, Memorial Health Institute, Aleja Dzieci Polskich 20, 04-736 Warszawa, Poland; 7 Department of Bone Marrow Transplantation, Pediatric Oncology and Hematology, Medical University of Wrocław, Bujwida 44, 50-368 Wrocław, Poland; 8 Department of Neuropathology and Molecular Pathology, Medical University of Gdańsk, Dębinki 7, 80-211 Gdańsk, Poland

## Abstract

**Background:**

TrkA (encoded by *NTRK1 *gene), the high-affinity tyrosine kinase receptor for neurotrophins, is involved in neural crest cell differentiation. Its expression has been reported to be associated with a favourable prognosis in neuroblastoma. Therefore, the entire coding sequence of *NTRK1 *gene has been analysed in order to identify mutations and/or polymorphisms which may alter TrkA receptor expression.

**Methods:**

DNA was extracted from neuroblastomas of 55 Polish and 114 Italian patients and from peripheral blood leukocytes of 158 healthy controls. Denaturing High-Performance Liquid Chromatography (DHPLC) and Single-Strand Conformation Polymorphism (SSCP) analysis were used to screen for sequence variants. Genetic changes were confirmed by direct sequencing and correlated with biological and clinical data.

**Results:**

Three previously reported and nine new single nucleotide polymorphisms were detected. c.1810C>T polymorphism present in 8.7% of cases was found to be an independent marker of disease recurrence (OR = 13.3; p = 0.009) associated with lower survival rates (HR = 4.45 p = 0.041). c.1810C>T polymorphism's unfavourable prognostic value was most significant in patients under 18 months of age with no *MYCN *amplification (HR = 26; p = 0.008). *In-silico *analysis of the c.1810C>T polymorphism suggests that the substitution of the corresponding amino acid residue within the conservative region of the tyrosine kinase domain might theoretically interfere with the functioning of the TrkA protein.

**Conclusions:**

*NTRK1 *c.1810C>T polymorphism appears to be a new independent prognostic factor of poor outcome in neuroblastoma, especially in children under 18 months of age with no *MYCN *amplification.

## Background

Neuroblastoma (NB), a tumour of the peripheral nervous system arising from embryonal neural crest cells (NCCs), is the foremost malignant neoplasm of neonate and infant [1]. It demonstrates considerable diversity in clinical behaviour, ranging from spontaneous regression or maturation to rapid progression despite aggressive therapy [2]. Among molecular factors influencing the clinical outcome of neuroblastoma, the expression of TrkA has been associated with favourable prognosis [3].

TrkA is a member of a Trk family of tyrosine kinase receptor for nerve growth factor (NGF) and other neurotrophins. TrkA primarily regulates growth, differentiation and programmed cell death of neurons in both peripheral and central nervous systems [4]. Formation of the neuronal network during maturation of the peripheral nervous system is a complex process that is rigorously governed by the interactions of neurotrophins with their respective Trk receptors [5]. Interestingly, TrkA expression may be involved both in regulation of cell differentiation and in induction of programmed cell death of NCCs sympathoadrenal lineage. Consequently, alterations in the genes of the Trk family may result in oncogenic transformation of the neural crest derivatives [6]. In NBs, the amount of NGF in the tumour microenvironment and expression of TrkA receptor has a profound effect on cellular behaviour [7]. Tumour cells expressing TrkA undergo cell differentiation in the presence of NGF [8], while withdrawal of NGF induces apoptosis. Finally, as previously reported, TrkA expression is a crucial factor for spontaneous regression of neuroblastoma [5].

Human TrkA coding gene (named *NTRK1*) maps to chromosome 1q21-q22 and is composed of 17 exons [9,10]. Neither germ-line nor somatic gain-of-function mutations of the *NTRK1 *gene have been observed in any human neoplasia [11-15], except for one case of a *de novo *AML. That patient was found to carry *NTRK1 *S677N mutation that might interfere with the tyrosine kinase (TK) activity [11]. A small number of polymorphisms of unknown physiological significance has been reported in a few cases of prostate cancer, papillary thyroid carcinoma and NB [13-15]. Inborn inactivating mutations of *NTRK1 *gene are responsible for neurodevelopmental abnormalities known as CIPA (Congenital Insensitivity to Pain with Anhidrosis; MIM#256800), a rare recessive genetic disease [16]. Interestingly, the clinical profile of CIPA does not include predisposition to cancer.

Although many publications provide information on the role of TrkA expression in NB [17], only one pilot study analysed partially *NTRK1 *gene sequence in NBs [13]. Therefore, we performed mutational analysis of the entire coding sequence of the *NTRK1 *gene to identify somatic mutations and/or polymorphisms which might alter TrkA expression and to evaluate clinical and biological consequences of their incidence for the patients with NB.

## Methods

Fifty-five NB patients treated in Polish paediatric oncologic centres and 114 NB patients retrieved from the Italian Neuroblastoma Group were enrolled into the study. Main clinical and biological characteristics of the patients, including age, stage of the disease, histology subtype in accordance with International Neuroblastoma Pathology Classification (INPC), *MYCN *amplification status and 1p36 chromosome deletion, are shown in Table 1. A control group of 158 healthy anonymous adult volunteers without history of hereditary cancer or other chronic disorders was included into the study on the basis of a detailed questionnaire and clinical examination. Written informed consent was obtained from the relevant guardians of the children and from patients and controls themselves, whenever eligible. The study was approved by the Ethics Committee of the Medical University of Gdańsk, Poland.

**Table 1:** Clinical and biological Features of Neuroblastoma Patients.

FACTOR	Dichotomous covariate	PL	IT	TOTAL
**AGE**	≤ 18 months of age	21 (38%)	76 (67%)	97 (57%)
	>18 months of age	34 (62%)	38 (33%)	72 (43%)

**STAGE**	1, 2, 3, 4S	28 (51%)	88 (77%)	116 (69%)
	4	27 (49%)	26 (23%)	53 (31%)

**Histological subtype (INPC)**	FH	17 (31%)	20 (18%)	37 (22%)
	UH	37 (67%)	88 (77%)	125 (74%)
	unknown	1 (2%)	6 (5%)	7 (4%)

***MYCN***	non-amplified	29 (53%)	92 (81%)	121 (72%)
	amplified	20 (36%)	22 (19%)	42 (25%)
	unknown	6 (11%)	0	6 (3%)

**1 p del/imbalance**	1 p normal	14 (25%)	52 (46%)	66 (39%)
	1 p deletion/imbalance	22 (40%)	38 (33%)	60 (36%)
	unknown	19 (35%)	24 (21%)	43 (25%)

Abbreviations: PL - Polish, IT - Italian group of patients; FH - favorable, UH - unfavorable histology: in accordance to International Neuroblastoma Pathology Classification (INPC)

DNA was extracted from fresh-frozen and/or paraffin-embedded tumour samples obtained during initial surgery procedures and from leukocytes of healthy controls. Tumour DNA was analysed because peripheral blood samples were not readily attainable from NB patients representing a number of oncologic centres from two countries. After digestion with proteinase K, DNA was purified by standard phenol-chloroform procedure [18]. All coding exons and flanking intronic (ca. 50 bp) sequences of *NTRK1 *gene were screened for mutations using two complementary techniques: SSCP (single-strand conformation polymorphism) analysis and DHPLC (denaturing high performance liquid chromatography). SSCP was performed in 12% polyacrylamide gels run at 200V for 2-4 hrs in 4°C followed by silver staining, as previously described [15]. DHPLC was performed on an automated WAVE System 3500 (Transgenomic Inc., San Jose, CA). Samples showing variation in SSCP banding pattern and/or abnormal profile of elution by DHPLC as well as a few randomly selected negative samples were subject to sequencing. DNA sequencing reactions were performed using capillary-based ABI 3100 Genetic Analyser (Applied Biosystems Inc., Foster City, CA).

Selected bioinformatic tools were used to assess the effect of sequence variants on the structure and function of the receptor. Alignment analysis of 4606 sequences similar to TrkA retrieved from Pfam database [19] (as for 11.2008) was used to identify conservative amino acid residues. Protein Data Bank [20] (last accessed 11.2008) was searched to identify the most structurally similar model of the TK domain, which was later used as a template for construction of the TrkA homology model. The fold recognition methods were used https://genesilico.pl/meta2/. The homology model was used to map mutated residues onto the three-dimensional structure of a prototypical kinase domain. Haplotype analysis of the entire *NTRK1 *gene sequence (chromosome 1q 155,097,295-155,118,266) was performed using data representative for the four distinct populations retrieved from International HapMap Project database [21] (http://hapmap.ncbi.nlm.nih.gov/ last accessed 05.2008) using Haploview software [22].

In all statistical analyses, allele frequencies were compared between case and control subjects within each population by chi-squared tests with adequate corrections, Fisher exact probability tests with Freeman-Halton extension for 2 × 3 tables and in the two populations combined by the Cochran-Mantel-Haenszel statistics. MAF (minor allele frequency) <5% was the criterion for exclusion from further statistical analysis. Unconditional logistic regression was used to examine the association between *NTRK1 *genotypes and clinical and biological characteristics of the disease. The dichotomous NB prognostic risk factors used as covariates are summarised in Table 1. The effect of the genotypes on long-time survival rates were analysed using Kaplan-Meier survival probability estimates, log-rank tests and Cox hazard proportional models. In the analysis of the two populations combined, an indicator variable for the study population (Poland versus Italy) was added to the models.

## Results

**Figure 1:**
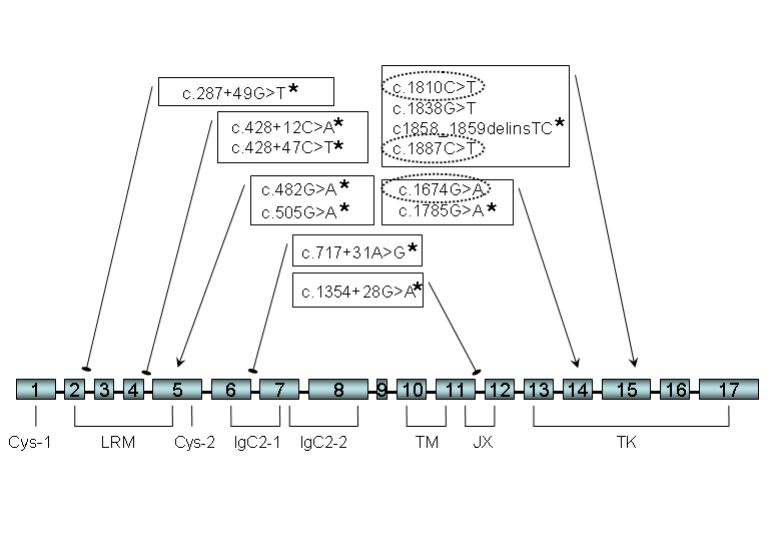
**Sequence variants of the *NTRK1 *gene identified in the present study**. The structure of TrkA receptor. Extracellular part of the protein consists of: two cysteine clusters (Cys); three leucine-rich motifs (LRM) and two immunoglobulin-like C2 domains (Ig-C2). It is followed by a short transmembrane portion (TM), and intracellular juxtamembrane domain (JM) and tyrosine kinase domain (TK). * - newly identified sequence variants, circled - the SNPs having MAF (minor allele frequency) value >5%, arrows - exonic localization of the variants, dishes - intronic localization of the variants.

Nine new *NTRK1 *gene variants were identified in NB samples. Four of them were located in the coding regions: c.482G>A, c.505G>A in exon 5; c.1785G>A, c.1858_1859delinsTC in exon 15 (Figure 1; Electronic supplementary material - additional file 1). Besides, three previously reported single nucleotide polymorphisms (SNPs) were detected [15-17]: c.1674G>A(rs6334), c.1810C>T(rs6336) and c.1887C>T(rs6337). (Figure 1; Electronic supplementary material - additional file 2). A nested study of the corresponding germline DNA confirmed presence of the SNPs at constitutional level.

**Figure 2:**
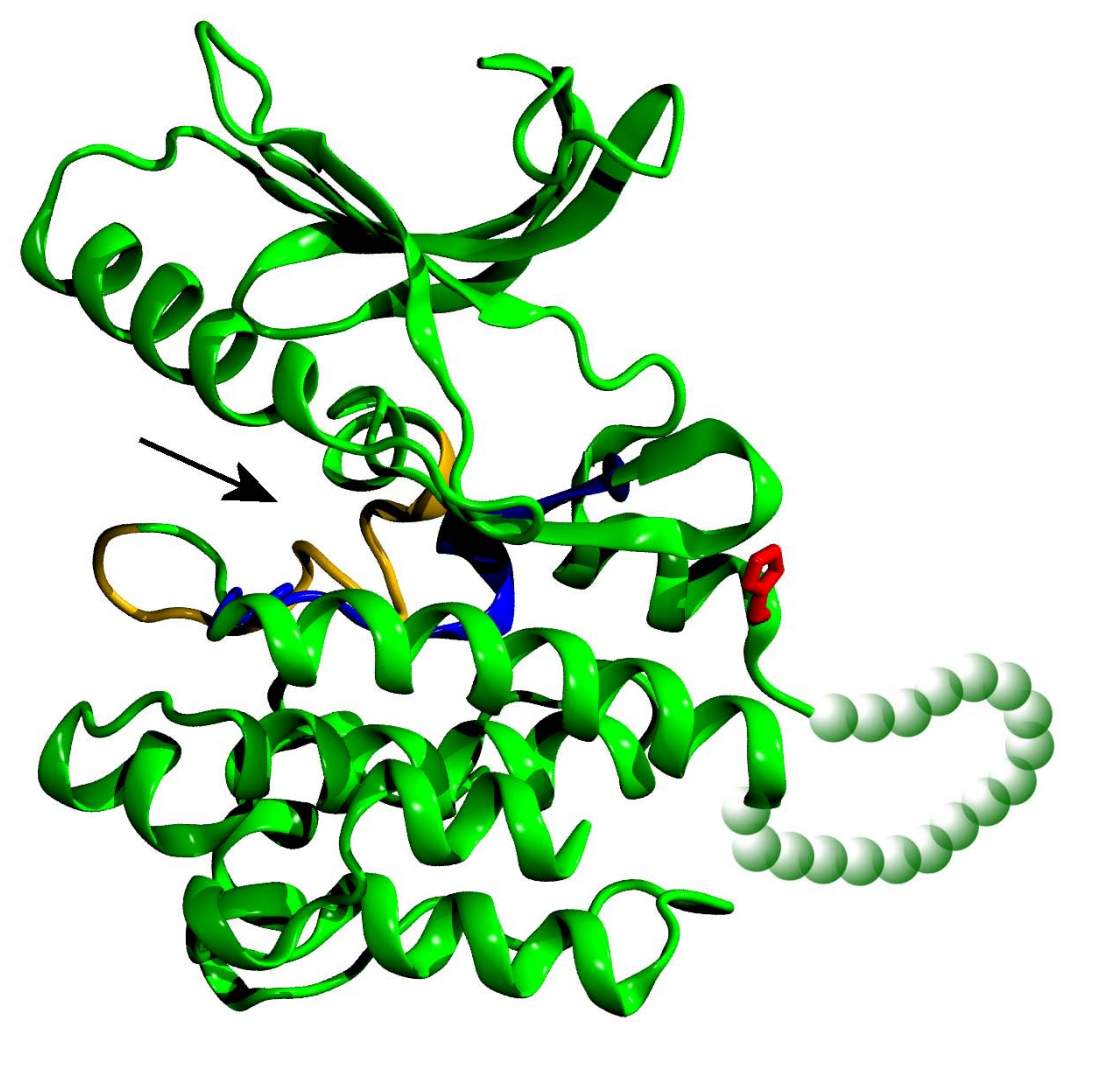
**Homology model of the TrkA TK domain**. The model was obtained using rat MuSK kinase structure *1luf *[23]. Activation loop (orange) and catalytic loop (blue) are indicated in accordance to de Pablo *et al. *[31]. The kinase insert fragment of kinase domain constitutes a loop joining two alpha-helices. Most of its residues are missing in the crystal structure presumably due to the loop's high conformational flexibility and therefore not present in homology model (depicted as green beads). The His604 residue (red) is located at the end of structurally determined helical region, distant from ATPase active site (arrow). It may, however, be involved in interactions with residues of the unstructured loop region. Although the function of this loop is not yet known, it may be speculated that it is involved in specific for TrkA interactions with secondary messengers or regulatory proteins. Homology model was visualized with VMD program [32].

For all non-synonymous variants (i.e. c.482G>A, c.505G>A, c.1785G>A, c.1810C>T, c.1858_1859delinsTC) *in silico *structure-function analyses have been performed. c.482G>A codes the last residue of the third leucine-rich motifs region. It is the last residue of the α-helix and the replaced amino acid has similar physical characteristics, thus the probability of its influence on protein structure is rather low. Similarly, c.505G>A most likely does not affect the protein structure, since it encodes a substitution of a residue in a non-structuralised fragment of the protein. The remaining three non-synonymous SNPs encode residues of the TK kinase, among which residue 604 (coded by c.1810C>T) is the only one evolutionarily conserved. We found an experimentally determined structure of MuSK TK *1luf *[23] to have the highest similarity with the TrkA TK domain. Sequence alignment revealed that all of the mutated TK residues lie in the kinase insert (TrkA residues 602-622) at the C-terminal lobe. This is a region of variable size and unknown function in many receptor TKs [23]. Homology modelling of TrkA TK domain, based on *1luf *structure file, allowed us to visualise the localisation of His604 relative to the domain's functional regions (Figure 2).

Haplotype analysis of the *NTRK1 *gene revealed 12 tag SNPs forming no haplotype blocks (Electronic supplementary material - additional file 3). 2 of 12 tag SNPs were exonic: c.1674G>A and c.1810C>T - and they were in moderate linkage disequilibrium (Lewontin coefficient D' = 1.0; LOD = 3.59; r^2 ^= 0.165; 2.6 kb distance). The presence of such disequilibrium was confirmed by our experimental data (D' = 0.568 [0.35-0.7]; LOD = 4.74; r^2 ^= 0.094).

SNPs characterised by MAF>5% were further evaluated to identify possible correlations with clinical and biological prognostic markers of the disease. Results of Cochran-Mantel-Haenszel analysis allowed the merging of data coming from two (Polish and Italian) patient populations. For each SNP heterozygotes and minor allele homozygotes were compared with major allele homozygotes frequencies. Genotype distribution did not deviate significantly from Hardy-Weinberg equilibrium. There were no statistically significant differences in MAF between patients and unaffected controls (Electronic supplementary material - additional file 2). Association study revealed no correlation between particular SNP and the incidence of NB. Spearman rank correlation analyses showed that none of the *NTRK1 *genotypes was associated with the presence of any of the evaluated NB prognostic markers. Logistic regression analyses identified c.1810C>T polymorphism as an independent statistically significant risk factor for disease recurrence (OR = 13.3; 95%CI: 1.9-94.0) along with *MYCN *amplification (OR = 9.79; 95%CI: 2.11-45.52) and clinical stage 4 (OR = 4.54; 95%CI: 1.08-19.1); p = 0.009.

**Figure 3:**
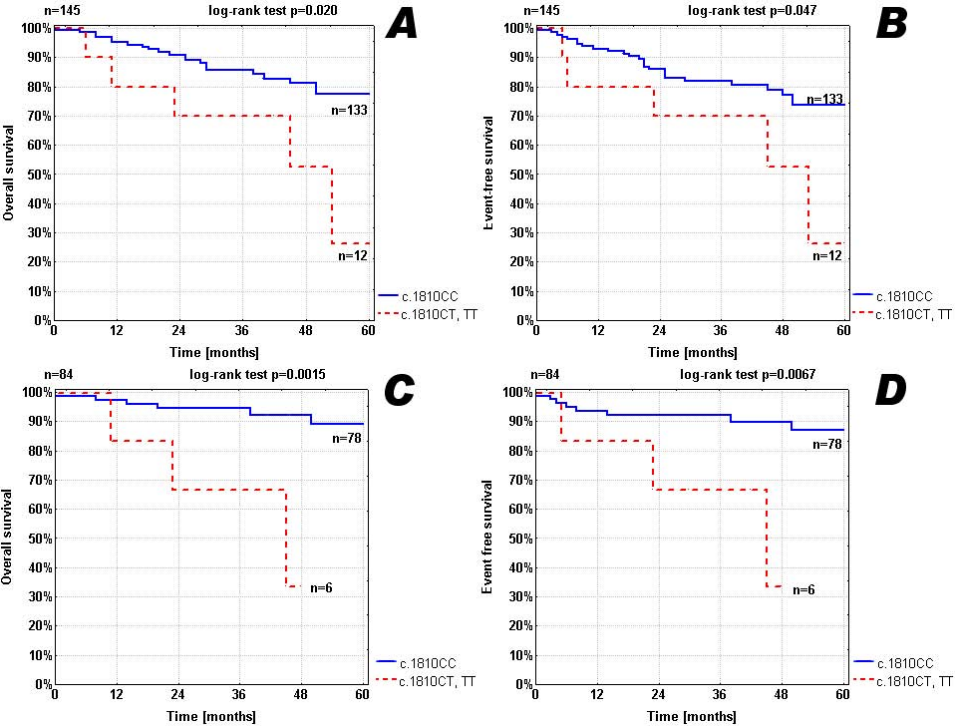
**Kaplan-Meier estimates of 5-year OS (A) and EFS (B) for NB patients and 5-year OS (C) and EFS (D) for NB patients under 18 months of age and without *MYCN *amplification with respect to c.1810C>T polymorphism**. Figures A and B show results for the entire group of NB patients (n = 145): 133 patients with *NTRK1 *c.1810CC genotype and 12 cases with *NTRK1 *c.1810CT/c.1810TT genotype. Figures C and D show results for the group of NB patients under 18 months of age and without MYCN amplification (n = 84). This subgroup consists of 78 patients with *NTRK1 *c.1810CC genotype and 6 cases with *NTRK1 *c.1810CT/c.1810TT genotype.

Overall (OS) and event free (EFS) survival rates were analysed for each of the SNPs separately using log-rank tests and Kaplan Meier plots. The presence of c.1810T allele was associated with shorter survival rates (Figure 3). Five-year OS for c.1810CC was 77.6% (95%CI: 68.6-86.6) v. 26.3% (95%CI: 0-66.8) for c.1810CT and c.1810TT (p = 0.020); while 5-year EFS were 73.8% (95%CI: 64.5-83.1) and 26.3% (95%CI: 0-66.8) (p = 0.047), respectively. Inferior survival rates related to the presence of c.1810T allele were even more evident in the group of children under 18 months of age and without *MYCN *amplification (Figure 3C, D). Here, 5-year OS was 89.2% (95%CI: 80.4-98.0) v. 33.3% (95%CI: 0-83.2) p = 0.0015; while 5-year EFS were 86.9% (95%CI: 77.8-96.0) and 33.3% (95%CI: 0-83.2) p = 0.0067, respectively.

The results of univariate analyses were further confirmed by Cox proportional hazard analysis. Firstly, a model inclusive of *NTRK1 *genotypes and adjusted for classical risk factors (7 variables - Table 2) was assessed. Three parameters were found to be significantly associated with shorter OS, comprising c.1810T allele having HR = 4.54 (95%CI: 1.07-19.3) at p = 0.041 (overall level of significance of the Cox model p = 0.00016). In the second step, backward elimination of features aimed at identification of minimal set of parameters predictive for OS was performed. This further confirmed c.1810T allele to be a significant marker of inferior OS, along with *MYCN *amplification and patient's age. Inclusion of the information on stage of the disease did not improve the model (data not shown). This finding is probably due to a strong association of stage and *MYCN *amplification (43% of patients with stage 4 harboured *MYCN *amplification in their tumours versus only 22% of patients with earlier stages; p = 0.001 by Fisher's exact test) and a strong association between stage and age <18 months (25% of patients with stage 4 were under 18 months versus 72% of patients with earlier stages; p = 0.006 by Fisher's exact test). We did not observe an association between *MYCN *amplification and c.1810T allele (p = 1.00 by Fisher's exact test) or between age and c.1810T allele (p = 1.00 by Fisher's exact test).

**Table 2:** Significance of the *NTRK1 *genotypes of MAF>5% and clinical and biological features of the disease on long-time OS rates assessed by multivariate Cox hazard regression model (overall significance of the model at p = 0.00016).

Feature	HR	95%CI	p value (Wald statistics)
Age ≤18 months	**0.22**	**0.07-0.70**	**p = 0.010**

*MYCN *amplification	**3.47**	**1.11-10.8**	**p = 0.032**

*NTRK1 *c.1810T allele	**4.54**	**1.07-19.3**	**p = 0.041**

unfavorable histology	3.01	0.67-13.4	p = 0.149

chromosome 1 p deletion/imbalance	1.44	0.46-4.46	p = 0.531

*NTRK1 *c.1887T allele	0.69	0.15-2.99	p = 0.619

*NTRK1 *c.1674A allele	1.21	0.43-3.43	p = 0.714

Stage 4	1.15	0.42-3.14	p = 0.789

HR - hazard ratio; CI - confidence intervals

Besides, in the group of patients under 18 months of age and without *MYCN *amplification, c.1810T was found to be the only independent prognostic parameter with HR = 26 (95%CI: 2.01-339); p = 0.008.

## Discussion

The basic alteration in NB is developmental arrest of neuronal differentiation and unrestricted proliferation that most probably result from altered expression of genetic factors. Studies of familial cases of NB allowed for identification of two NB susceptibility genes (*ALK*, *PHOX2B*) yet their mutations are present in about 10% of sporadic tumours [24,25]. Inability to identify a single major specific tumour suppressor gene as a triggering agent of NB initiation [2] points toward the role played by genes involved in normal noradrenergic development. *NTRK1 *gene encoding TrkA neurotrophin receptor for NGF, is referred to as a candidate predisposition gene [5]. In this study, we investigated the genetic variability at the *NTRK1 locus *in human NBs.

Previously, only one study analysed sequence of *NTRK1 *gene in NBs [13] and that analysis was limited to the region coding TK domain (exons 13-16). This and two other studies of the *NTRK1 *sequence: in prostate cancer [15] and medullary thyroid carcinoma [14], utilised SSCP as the sole screening method. However, current standards of molecular diagnostics do not recommend SSCP as a single screening procedure for detection of sequence variants because of its low sensitivity of 50-86%, while DHPLC is considered to be the method of choice because of its sensitivity exceeding 99% [26]. In our study, we used both methods. DHPLC vastly outperformed SSCP, allowing for detection of a notably larger number of sequence variants (11/12 versus 5/12 SNPs identified).

Although TrkA expression in NBs was reported significantly variable even at the cellular level within a single case [27], the genetic variability at the *NTRK1 *gene *locus *was found to be very limited. Non-synonymous polymorphisms were detected in less than 10% of the analysed cases. Furthermore, tag SNPs identified by HapMap project as well as SNPs present in NCBI dbSNP in vast majority are located in the intronic sections of the gene or do not result in amino acid substitution. Besides, according to the results of bioinformatic analyses, the identified non-synonymous polymorphisms, with the exception of c.1810C>T, have minimal theoretical chance of affecting the protein's structure and/or functions. These findings confirm the conservative character of *NTRK1 *gene sequence; however, it is possible that significant sequence variants are located elsewhere, for instance in currently unknown 3' flanking regulatory elements.

Even though c.1810C>T has MAF of 6%, from comprehensive statistical analysis of the associations with NB clinical and biological prognostic factors it appears to be an independent unfavourable risk factor in NB patients. c.1810T allele has been detected in 8.7% of sporadic tumours, which is a similar frequency to the mutations observed for the two known familial NB susceptibility genes (*ALK *= 12.4%, *PHOX2b *= 4.3%) [24,25]. Presence of c.1810T allele correlated with inferior 5-year OS and EFS and with more frequent relapse of the disease. From the analyses of the Cox hazard regression models, it appears that c.1810T provides additive information to *MYCN *amplification status and age of the patient, allowing for finest prediction of the eventual outcome of the disease. Interestingly, the stage of the disease was less informative and could be omitted in the model based on minimal set of predictive parameters. The lack of prognostic significance of stage is most probably due to strong associations with other prognostic variables - *MYCN *amplification and age. These associations were not observed for the *NTRK1 *c.1810C>T polymorphism.

The additive value of c.1810T allele to the current clinical prognostic markers should be, however, assessed on a prospective, more coherent, group of patients. In the current study, patients were enrolled in two countries over 20 years, thus they received a range of treatment protocols. Also, histopathological assessment was modified over the time and not all the samples could be assessed in accordance with the revised INPC. Interestingly, IT patients were diagnosed with lower stages of the disease and at a younger age, which is most probably related to better access to medical imaging techniques in Italy and thus higher incidence of incidentalomas diagnosed before overt clinical symptoms of advanced disease became apparent. The above-presented discrepancy in the patient population and the fact that the study was retrospective might, however, result in some limitation of the force of the study.

The mechanisms through which c.1810T variant could trigger and/or modify oncogenic events in embryonal NCC are only hypothetical at this time; however, our preliminary data provide an interesting starting point for further studies, possibly using a genome-wide association methodology.

Several points strongly support the significance of our finding; a) first, the evidence of the observed correlations was highly significant, both in univariate and multivariate analyses. Similarly, in the previous study of 63 NBs [13], there was a trend for the c.1810T to be present in cases having advanced stages of the disease; b) there were no major differences in allelic frequencies among two European populations that would explain these findings on the basis of population stratification. Linkage disequilibrium between c.1674G>A and c.1810C>T was present in both groups of patients, and also in four populations from HapMap database, thus the results obtained in both patient groups most probably refer to the same genetic effect; c) the bioinformatic analysis of the c.1810C>T showed that substitution of the corresponding amino acid (His→Tyr) affects a conservative residue of the TK domain. This substitution changes the chemical properties of a residue in conserved position, which may possibly alter the function of TK domain. From analysis of our homology model as well as from the results of *in vitro *studies by Mardy *et al*. [28], it appears that c.1810C>T does not inhibit the ability for autophosphorylation of TK domain. It may, however, be involved in interactions with the residues of the kinase insert loop. The exact role of this fragment of TK, specific for TrkA, remains unknown though it may be involved in signal transduction through interfering with the adaptor molecules.

Finally, a few methodological considerations regarding peculiarities of gene association studies performed on a paediatric oncologic experimental group should be pointed out. The relatively low incidence of neuroblastoma of 10.9 cases per million children yearly [29] affects the available size of patient sample group. In order to overcome this limitation, patients representing two European populations were enrolled in the study after initial validation of their correspondence using the Cochran-Mantel-Haenszel analysis. Another important methodological issue is related to the design of an adequate control group. If age-matched child controls had been used in the study, a screening for clinically inert tumour would have been required, however this might have created an ethical dilemma. Therefore, similarly to other studies in the field [30], healthy adult volunteers were chosen as controls, even if such a decision was related to some decrease in the force of the study. Moreover, even though the study was aimed at detection of constitutional mutations, since the available material collected by both national tissue banks consisted of primary NB tumours, tumour DNA was analysed. A similar approach is applied in studies of other researchers [24,25] because tumour biology is the area of primary interest from the clinical point of view. Besides, tumour samples are the routine material available for molecular assessment in research setting. Nevertheless, supplementary nested study of the corresponding germline DNA, derived from peripheral blood leukocytes of the cases where such material was available, was performed to provide confirmation of the constitutional character of the analysed SNPs.

## Conclusions

We propose *NTRK1 *c.1810C>T polymorphism as a new prognostic factor in NB. In contrast to known NB susceptibility genes (*ALK *and *PHOX2b*), *NTRK1 *c.1810C>T polymorphism does not predispose to NB development. Conversely, its presence confers worse prognosis to the affected patients. Nevertheless, further studies are needed to disclose its effect on TrkA receptor structure and/or function, for instance by comparing the expression levels of wild type and mutated TrkA at RNA and protein levels.

## Competing interests

None of the authors declare any financial and personal relationships with other people or organizations that could inappropriately influence (bias) the work. This includes employment, consultancies, stock ownership, honoraria, paid expert testimony, patent applications/registrations, and grants or other funding.

## Authors' contributions

BL conceived of the study, carried out the molecular genetic studies, performed the statistical analysis, participated in the sequence alignment and drafted the manuscript. ED participated in the design of the study and its coordination and provided clinical data. PS carried out the molecular genetic studies. GP coordinated the study of the Italian patients and helped to draft the manuscript. SZ performed *in-silico *analyses and helped to draft the manuscript. EIS, DP, AC provided tumor samples and clinical data and coordinated the study of the Polish patients. WB participated in statistical analysis and helped to draft the manuscript. JL conceived of the study, participated in its design and coordination and helped to draft the manuscript. All authors read and approved the final manuscript.

## Pre-publication history

The pre-publication history for this paper can be accessed here:

http://www.biomedcentral.com/1471-2407/9/436/prepub

## Supplementary Material

Additional file 1 **The identified new sequence variants of the *NTRK1 *gene**. Detailed list of all (exonic and intronic) new sequence variants of the *NTRK1 *gene identified in the study. Click here for file

Additional file 2 **The frequencies of the common (MAF>5%) sequence variants of the *NTRK1 *gene in the two groups of patients and the healthy controls**. Details regarding frequencies of the SNPs with MAF>5% in the analyzed populations and the control group along with the results of the Hardy-Weinberg equilibrium analysis. Click here for file

Additional file 3 **Haplotype analysis of the SNPs representative for *NTRK1 *locus based on the data retrieved from the HapMap Project database**. Results of the haplotype analysis of the SNPs representative for *NTRK1 *locus based on the data retrieved from the HapMap Project database. The analysis was performed using Haploview software [22]. In the upper part of the scheme physical map of the corresponding fragment of the chromosome is given, the middle part of the scheme shows exact intronic/exonic localization of the SNPs and the lower part illustrates identified associations. (rs6334 - c.1674G>A; rs6336 - c.1810C>T). Color code reflects the strength of association: **WHITE COLOR**: D' < 1, LOD<2 - insignificant; **BLUE COLOR**: D' = 1 LOD<2 high degree of recombination; **INTENSIVITY OF THE RED COLOR**: D' < 1; LOD = 2 reflects the strength of association, the maximum of intensity is reached at D' = 1, LOD = 2 - high level of association. The numbers on the diamonds show D' value multiplied by 100. Click here for file
