# Supplementary material for: c.1810C>T Polymorphism of NTRK1 Gene is associated with reduced Survival in Neuroblastoma Patients
Source: BMC Cancer. 2009 Dec 13;9:436. doi: 10.1186/1471-2407-9-436 (PMC2800120; doi:10.1186/1471-2407-9-436)
Supplement: Additional file 1 — The identified new sequence variants of the NTRK1 gene. Detailed list of all (exonic and intronic) new sequence variants of the NTRK1 gene identified in the study. [file 1471-2407-9-436-S1.PDF]

Additional file 1. The identified new sequence variants of the *NTRK1* gene.

Codon /	Nucleotide/	Exon	Allele	Amino acid residue	Number of cases
I	287+49	2-3	G>T	none	2
I	428+12	4-5	C>A	none	1
I	428+47	4-5	C>T	none	1
161	482	5	G>A	Arg → His	1
169	505	5	G>A	Gly → Arg	1
I	717+31	6-7	A>G	none	1
I	1354+28	11-12	G>A	none	1
595	1785	14	G>A	Gly	1
620	1858-59	15	GG>TC	Gly → Ser	2

I - intronic localization of the sequence variant.
