# Supplementary material for: c.1810C>T Polymorphism of NTRK1 Gene is associated with reduced Survival in Neuroblastoma Patients
Source: BMC Cancer. 2009 Dec 13;9:436. doi: 10.1186/1471-2407-9-436 (PMC2800120; doi:10.1186/1471-2407-9-436)
Supplement: Additional file 2 — The frequencies of the common (MAF>5%) sequence variants of the NTRK1 gene in the two groups of patients and the healthy controls. Details regarding frequencies of the SNPs with MAF>5% in the analyzed populations and the control group along with the results of the Hardy-Weinberg equilibrium analysis. [file 1471-2407-9-436-S2.PDF]

Additional file 2 The frequencies of the common (MAF>5%) sequence variants of the *NTRK1* gene in the two groups of patients and the healthy controls.

SNP	Group	Frequency of heterozygotes	p value for Hardy-Weinberg equilibrium	Percentage of genotypes [%]	MAF	Alleles	Difference in SNP frequency between patients (PL+IT) and unaffected controls
c.1674G>A	PL	0.245	0.7781	96.4	0.160	G:A	p= n.s.
	IT	0.279	0.8632	97.4	0.158		OR=0.67
	PL+IT	0.262	-	96.9	0.159		95%CI 0.41-1.09
	CONTROL	0.336	0.9996	82.4	0.214		
c.1810C>T	PL	0.077	0.1023	94.5	0.058	C:T	p= n.s.
	IT	0.082	0.2989	86.0	0.051		OR=0.73
	PL+IT	0.080	-	90.3	0.055		95%CI 0.33-1.62
	CONTROL	0.124	0.7956	66.0	0.062		
c.1887C>T	PL	0.350	0.3015	100.0	0.172	C:T	p= n.s.
	IT	0.300	0.7592	24.0	0.220		OR=0.71
	PL+IT	0.325	-	62.0	0.196		95%CI 0.41-1.25
	CONTROL	0.390	0.7069	85.5	0.240		

Abbreviations: PL – Polish, IT- Italian group of patients; MAF – minor allele frequency; n.s. – non significant; OR –odds ratio; CI -95% confidence intervals.
