# Supplementary figures and images for: c.1810C>T Polymorphism of NTRK1 Gene is associated with reduced Survival in Neuroblastoma Patients
Source: BMC Cancer. 2009 Dec 13;9:436. doi: 10.1186/1471-2407-9-436 (PMC2800120; doi:10.1186/1471-2407-9-436)

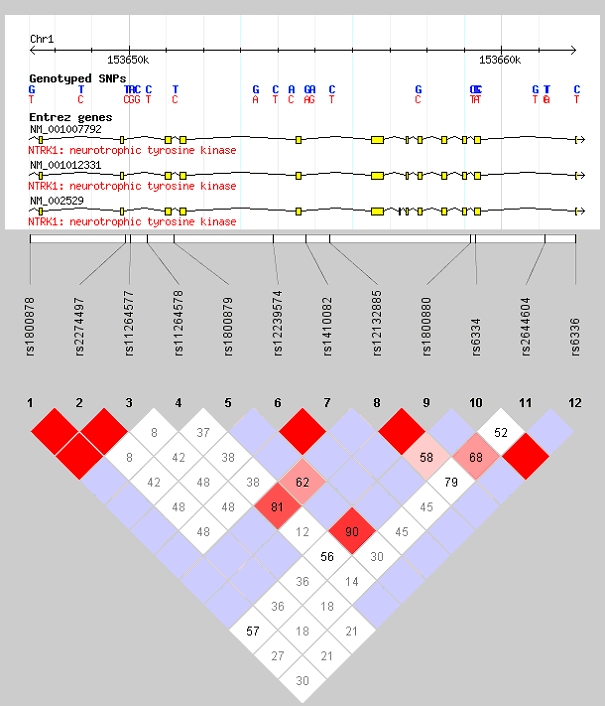

Supplement: Additional file 3 — Haplotype analysis of the SNPs representative for NTRK1 locus based on the data retrieved from the HapMap Project database. Results of the haplotype analysis of the SNPs representative for NTRK1 locus based on the data retrieved from the HapMap Project database. The analysis was performed using Haploview software [22]. In the upper part of the scheme physical map of the corresponding fragment of the chromosome is given, the middle part of the scheme shows exact intronic/exonic localization of the SNPs and the lower part illustrates identified associations. (rs6334 - c.1674G>A; rs6336 - c.1810C>T). Color code reflects the strength of association: WHITE COLOR: D' < 1, LOD<2 - insignificant; BLUE COLOR: D' = 1 LOD<2 high degree of recombination; INTENSIVITY OF THE RED COLOR: D' < 1; LOD = 2 reflects the strength of association, the maximum of intensity is reached at D' = 1, LOD = 2 - high level of association. The numbers on the diamonds show D' value multiplied by 100. [file 1471-2407-9-436-S3.JPEG]
